# Measuring Vulnerable Population's Healthy and Unhealthy Food Access in Austin, Texas

**DOI:** 10.3934/publichealth.2016.4.722

**Published:** 2016-09-12

**Authors:** Junfeng Jiao

**Affiliations:** Urban Information Lab, School of Architecture, The University of Texas at Austin, Austin, Texas, USA

**Keywords:** food desert, food accessibility, Geographic Information System

## Abstract

Food deserts—areas with a significant low-income population experiencing low accessibility to healthy food sources—have been well studied in terms of their connection to obesity and its related health outcomes. Measuring food accessibility is the key component in food desert research. However, previous studies often measured food accessibility based on large geographic units (e.g. census tract, zip code) with few transportation modes (e.g. driving or taking public transit) and limited vulnerable population measures. This paper aims to demonstrate a new method to measure food access for different vulnerable population groups at a smaller geographic scale with different transportation modes. In detail, this paper improves on previous studies from the following three perspectives: (1) Measuring food accessibility with a smaller geographic scale: block group vs. census track which on average includes 1000 people vs. 4000 people; (2) Measuring food accessibility with different transportation modes: walking, biking, transit, and driving vs. driving only; and (3) Measuring food accessibility for different vulnerable population groups. The proposed method was tested in the city of Austin, which is the capital of Texas and the 11th largest city in the US, and measured people's accessibility to both healthy and unhealthy food sources within the city. The methods can be applied to address food accessibility issues in other cities or regions.

## 1. Introduction

Food deserts—areas with a significant low-income population experiencing low accessibility to healthy food sources—have been well-studied in terms of their connection to obesity and its related health outcomes. Food access as the key component in food desert studies has traditionally been measured as the physical distance between the centroids of spatial units of analysis (e.g., census tracts or the 1-km grid as the neighborhood), or between the centroids of spatial units housing the population and the closest supermarket or large grocery store.

The methodological limitations of past studies included the use of coarse levels of income data aggregation, such as zip codes or census tracts, which could overlook the stronger demand for healthy food from smaller geographic areas. Second, vulnerable populations were usually measured based on few methods and there were limited comparisons of the final findings. Third, most studies focused on driving as the default transportation mode. These limitations traditionally led to vague, rough and even inaccurate food desert identification. The proposed study sought to improve previous food access research by demonstrating a GIS-based method quantifying different transportation food access for different vulnerable groups at a smaller geographic unit (block group), which is smaller than census tracts and typically has a population of 600 to 3,000 people [1].

## 2. Methods

### Data Collection and Analysis

Three categories of spatial data were utilized to perform a food accessibility analysis in Austin: vulnerable population data; food establishments; and transportation networks. Vulnerable populations were identified at the census block group level using the 2010 US Census and 2012 American Community Survey data [1],[2] and were based on the following four criteria: poverty rate greater than or equal to 20 percent; median family income not exceeding 80 percent of metro-area median family income; at least 40 percent at or below double poverty level; and, more than 30 percent without access to personal vehicle [3]–[6].

**Figure 1.**
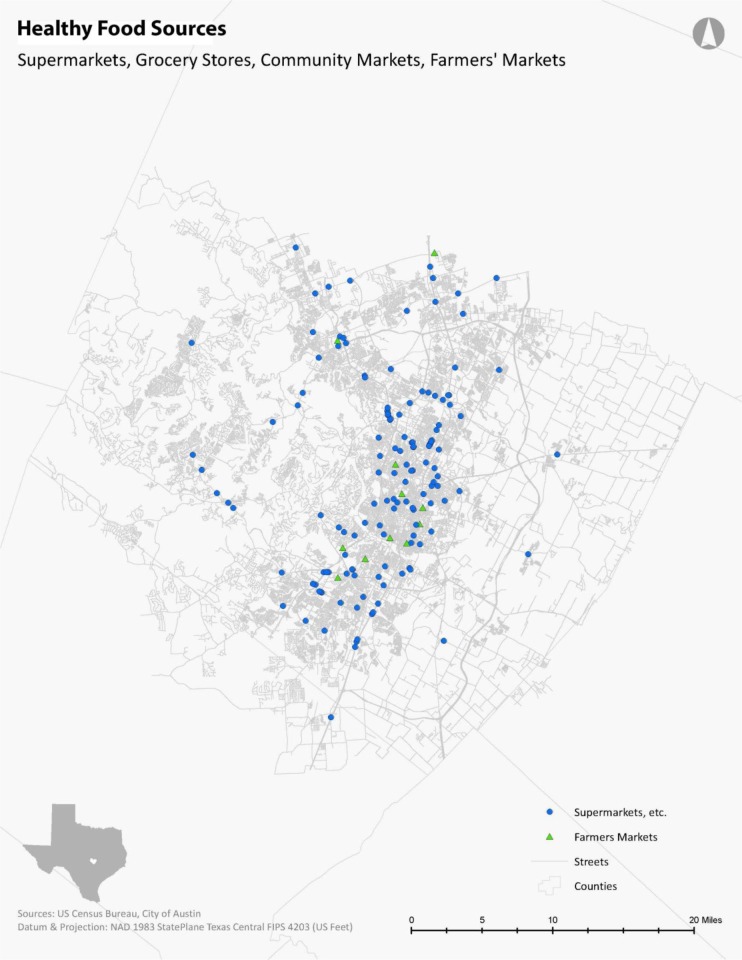
Healthy Food Source Locations in Travis County, Texas.

**Figure 2.**
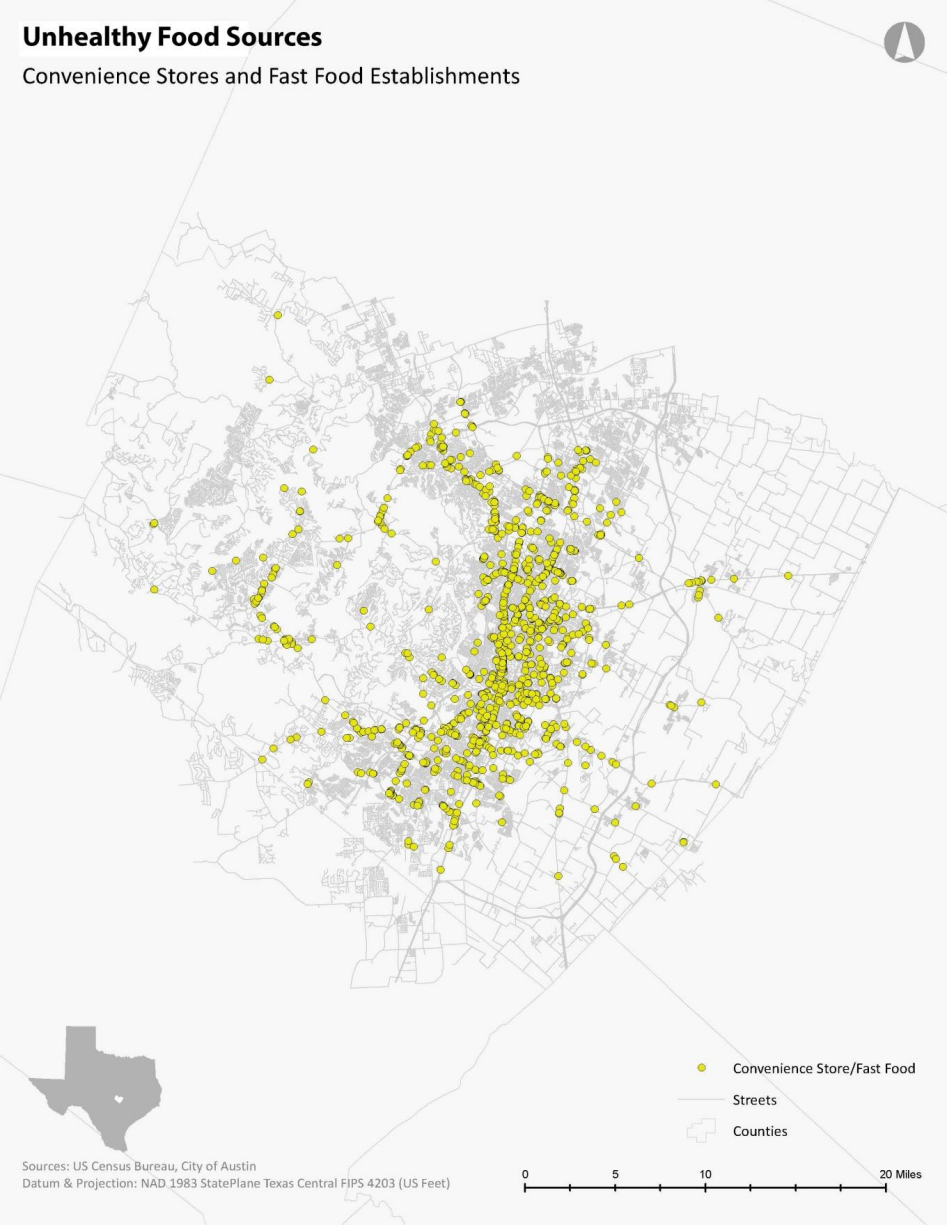
Unhealthy Food Source Locations in Travis County, Texas.

The food establishment permit data was collected from the City of Austin Department of Public Health. The dataset lists each food source by name, location, and detailed classifications (e.g. grocery store, supermarket, convenience store, etc.). Based on previous research, these food establishments were classified as healthy food sources (supermarkets/grocery stores, and farmers' markets) and unhealthy food sources (fast food restaurants and quick service restaurants, convenience stores, corner stores) [5],[7]. The accuracy of data for both healthy food sources and unhealthy food sources were verified through Google Maps. In addition, unlisted large-scale food sources (e.g. supermarkets) were identified by cross-referencing published store locations with the permitted food establishment dataset. These healthy and unhealthy food establishments were geocoded in GIS based on the permitted addresses (Figures 1 and 2).

Austin transportation network GIS data was collected from the Austin GIS and Maps Department and the Capital Metropolitan Transportation Authority. The dataset contained information regarding streets, bicycle infrastructure, sidewalks, transit routes and stops. Using the above Austin transportation network data, four separated GIS transportation networks were built for motor vehicle, bicycle, transit, and pedestrian routes in ArcGIS. The automobile network was generated using the complete City of Austin street network shapefile. The bicycle network excluded highways, freeways, and on/off-ramps from the City of Austin streets shapefile. Pedestrian street network—defined as surface streets with sidewalk infrastructure or a street with a speed limit no greater than 35 miles per hour—were also generated using the City of Austin streets shapefile. The transit network was established by using a modified Capital Metro transit route and stop shapefile. The travel time between stops was calculated by using the average route circulation times as published in the Capital Metro schedule book [8] and cross-verified with Google Transit data in the City of Austin.

Using the ArcGIS Network Analyst tool, ten-minute network buffers were generated for all City of Austin food establishments in each transportation network. Time impedance was used for the automobile and transit network service zones. The transit network buffer incorporated up to a half-mile walk along the pedestrian network as a requirement for transit accessibility [9],[10]. A distance proxy was decided to represent the bicycle and pedestrian network buffer: ten minutes of travel was represented by either a two-mile bicycle ride or a half-mile walk [11]. The combination of the individual transportation networks created the overall transportation service areas for both healthy and unhealthy food establishments in the City of Austin.

## 3. Results

The City's auto-centric Land Development Code has designated Austin into a city dependent upon the personal motor vehicle. Therefore, as expected, owning a vehicle in Austin, Texas, provides high levels of access to any food source. Regardless of the selected vulnerability indicator examined, more than 95% of any given population has access to both healthy and unhealthy food sources. A clear pattern emerges when focusing on the alternative transportation methods. Biking provides the second highest level of accessibility to healthy and unhealthy food sources regardless of the vulnerable population indicator. Transit provides the third highest level of accessibility to healthy and unhealthy food sources regardless of the vulnerable population indicator. Walking provides the lowest level of accessibility to healthy and unhealthy food sources regardless of the vulnerable population indicator. Because there are many more unhealthy food sources than healthy food sources in the city (Figure 1 and 2), as a result, unhealthy food sources are much more accessible by alternative travel modes than healthy food sources in Austin. Vulnerable populations can walk to almost three times more unhealthy food stores than to healthy food sources (Table 1).

**Table 1.** Transportation Access to Healthy and Unhealthy Food Establishments in Austin, TX.

**Table:** (a) Percent of Block Group Population with Access to Healthy Food Establishments.

*Vulnerable Population by Block Groups*	*Combined Population*	*Walk*	*Bike*	*Transit*	*Drive*
≥ 40% of Block Population Below Double Poverty Level	431,704	18%	76%	67%	96%
≥ 20% of Block Population Below Poverty Level	353,307	21%	82%	77%	98%
Block Median Family Income≤ 80% of Travis County MFI	376,045	21%	85%	78%	99%
≥ 30% without motor vehicle	9,400	37%	100%	94%	100%

**Table:** (b) Percent of Block Group Population with Access to Unhealthy Food Establishments.

*Vulnerable Population by Block Groups*	*Combined Population*	*Walk*	*Bike*	*Transit*	*Drive*
≥ 40% of Block Population Below Double Poverty Level	431,704	48%	83%	76%	96%
≥ 20% of Block Population Below Poverty Level	353,307	55%	90%	85%	98%
Block Median Family Income≤ 80% of Travis County MFI	376,045	55%	89%	85%	99%
≥ 30% without motor vehicle	9,400	91%	100%	100%	100%

Note: Transportation access to healthy and unhealthy food sources means that residents can be reached within ten minutes from the selected food source using the selected transportation mode.

**Figure 3.**
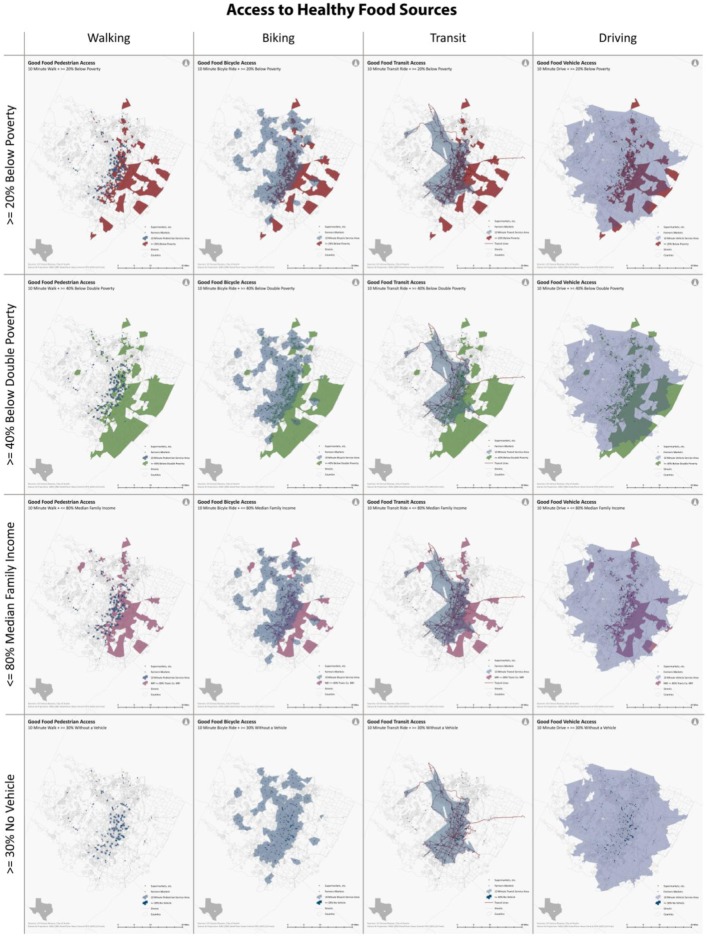
Transportation Access to Healthy Food Sources in Austin, Texas.

**Figure 4.**
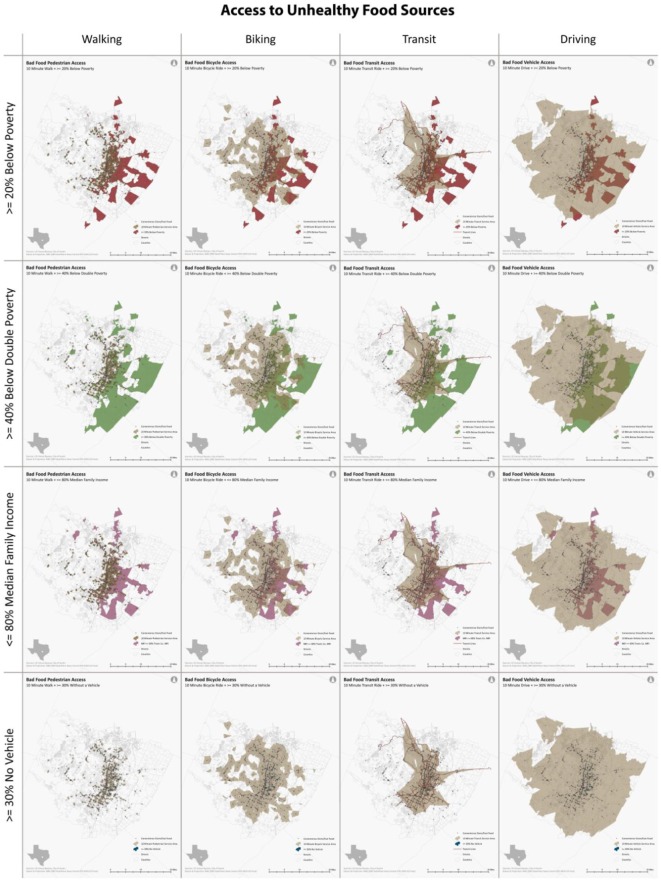
Transportation Access to Unhealthy Food Sources in Austin, Texas.

Census block groups with more than 40% of the population falling below the double poverty line had the lowest levels of access to both healthy and unhealthy food sources by all transportation methods. Food access was relatively equivalent for block groups classified as either “≥20% of block group population below poverty level” or “block median family income ≤80% of Travis County median family income” for all transportation methods. Block groups where at least 30% of the population (in households) did not have access to a motor vehicle had the greatest levels of food access. This might be because fewer neighborhoods satisfied this criterion compared to the other three vulnerable measures. Only about 9,400 people lived in these low car ownership neighborhoods compared to vulnerable populations of 350,000 to 430,000 identified by the other three criteria. Such differences can be clearly observed in figures 3 and 4, where the first three criteria identified much more vulnerable population groups and covered much larger areas (red, green, pink) than the fourth method (blue) (Figures 3 and 4).

## 4. Discussion

Food deserts and healthy food accessibility represent a supply-side issue (lack of healthy food sources) within a demand-side problem (citizens' access to food sources). The lack of a comprehensive, consensus method to measure food accessibility is thwarting attempts to implement public and land use policies in order to combat the problem. This research improved the previous major food accessibility measures [5],[12] from the following perspectives.

### 4.1. Unit of Analysis and Origin of Buffers

Previous food desert research often measured healthy food accessibility at the census tract level and used the “distance from the centroid to a given healthy store” [4],[5],[13] to represent food accessibility. Although the census tract is useful for providing census data regarding economically at-risk populations, the lack of regularity in the size and shape of census boundaries does not lend well to representing spatial data issues. The inconsistent and irregular sizes and shapes of census tracts can result in over identification or under identification of food deserts. To guard against this potential error, it is better to generate network buffers from each food source rather than from individual census tract centroids and measure vulnerable populations at the block group or smaller scale.

### 4.2. Mode of Access

Although the motor vehicle is the primary form of transportation in the United States and more than 90 percent of workers commute to work in privately owned cars [14], it is still important to measure food accessibility with alternative transportation modes. Not all are able to drive and vulnerable populations are more likely to rely on transit or other transportation modes for grocery shopping [15],[16]. To better measure people's access to different food sources, different transportation buffers were generated based on walking, biking, transit, and driving.

### 4.3. Different Vulnerable Population Definitions

This study identifies vulnerable populations based on different criteria used in previous studies and measured access to healthy and unhealthy food sources for these different population groups. By varying vulnerable population definitions, this research added one more dimension to food desert identifications and helped researchers to compare and contrast food access results and helped government to better access food desert problems in a given city or region.

## 5. Conclusion

This paper demonstrated a method to measure people's access to food sources with different transportation modes. Using the block group as the unit of analysis helps researchers to better identify vulnerable populations and neighborhoods. Establishing network buffers based on different transportation networks originating from food sources can better capture people's true access to healthy and unhealthy food sources. Both public health and urban planning benefit from more accurate spatial analysis techniques, which can better determine people's access to different food sources. Varying vulnerable population definitions and understanding how different transportation modes impede food access for vulnerable populations will allow planners to better allocate transportation resources to the most needed areas.
